# Cerebral Hemodynamic Changes After Endovascular Recanalization of Symptomatic Chronic Intracranial Artery Occlusion

**DOI:** 10.3389/fneur.2020.00318

**Published:** 2020-05-05

**Authors:** Kaijiang Kang, Bo Yang, Xiping Gong, Xing Chen, Weibin Gu, Guofeng Ma, Zhongrong Miao, Xingquan Zhao, Ning Ma

**Affiliations:** ^1^Department of Neurology, Beijing Tiantan Hospital, Capital Medical University, Beijing, China; ^2^China National Clinical Research Center for Neurological Diseases, Center of Stroke, Beijing Institute for Brain Disorders, Beijing, China; ^3^Department of Interventional Neuroradiology, Beijing Tiantan Hospital, Capital Medical University, Beijing, China; ^4^Department of Neurology, Beijing Jiangong Hospital, Beijing, China; ^5^Beijing Advanced Innovation Center for Biomedical Engineering, Beihang University, Beijing, China; ^6^Department of Radiology, Beijing Tiantan Hospital, Capital Medical University, Beijing, China

**Keywords:** stroke, intracranial occlusion, recanalization, hemodynamics, CT perfusion

## Abstract

**Objective:** We performed this study to evaluate the hemodynamic changes over time after successful endovascular recanalization in patients with symptomatic chronic intracranial artery occlusion (CIAO).

**Materials and Methods:** We included 20 patients with symptomatic CIAO in a high-volume stroke center from June 2014 to June 2019. All subjects were evaluated with CT perfusion (CTP) studies before and after the recanalization. The relative cerebral blood flows (rCBFs) in perforating artery territory (PAT) and cortical artery territory (CAT) of occluded arteries were compared before and after the recanalization. The patients were categorized into subgroups based on the time interval from revascularization to post-procedural CTP, occlusion sites, and restenosis status. The proportion of rCBF change (rCBFc%) was compared in variable subgroups.

**Results:** The rCBF increased significantly from 0.52 to 0.71 in PAT (*P* < 0.001) and from 0.59 to 0.85 in CAT (*P* < 0.001) after recanalization, and there were also statistical differences in variable subgroups except for those with restenosis. The median and interquartile range (IQR) of rCBFc% were 35.2 and 18.6–56.6%. For patients with short-term follow-up (55.2%), the rCBFc% was relatively higher than that in patients with mid-term (35.4%) and long-term follow-up (32.7%), although without statistical difference (*P* = 0.273). For patients with restenosis, the rCBFc% was significantly lower than that in patients without restenosis (18.5 vs. 37.3%, *P* = 0.008).

**Conclusions:** In patients with symptomatic CIAO, the CBF may increase and be relatively stable over time after successful recanalization except for restenosis.

## Introduction

Stroke has now become the first most common and disabling disease in China, and intracranial artery stenosis or occlusion is one of the leading causes of ischemic stroke in Asian populations (1, 2). Chronic intracranial artery occlusion (CIAO) is the extreme phase of intracranial atherosclerotic stenosis, and patients with CIAO have a higher risk of stroke recurrence due to hemodynamic compromise. It is reported that in patients with CIAO, the annual recurrence rate of stroke was 7.27% and can be as high as 23.7% in those with significant hypoperfusion (3, 4).

Given the high risk of recurrent stroke in patients with CIAO, endovascular angioplasty and stenting have been performed in clinical practice and some studies (5–10). Endovascular recanalization is supposed to increase cerebral perfusion, thereby reducing the risk of recurrent ischemic stroke and improving neurological outcome. Given the establishment of collateral circulation associated with chronic occlusions, it remains uncertain whether the CBF increases and what is the tendency of CBF change over time after successful recanalization. Previous studies had suggested that CT perfusion (CTP) imaging provides several radiological markers (especially CBF) to assess cerebral hemodynamic changes after successful revascularization in patients with chronically occluded cervical internal carotid artery (ICA), and improvement in CBF is associated with significant improvement in neurocognitive functions (11–13). We conducted this study to evaluate the hemodynamic changes over time after successful recanalization in patients with CIAO.

## Materials and Methods

### Study Population

The study was performed according to the guidelines from the Helsinki Declaration, and it was approved by the institutional review board (IRB) of the hospital. Written informed consent was obtained from all patients or their legally authorized guardian. We retrospectively and consecutively collected patients who were diagnosed with CIAO, received endovascular recanalization, and underwent whole-brain CTP before and after the recanalization in our stroke center from June 2014 to June 2019. The data that support the findings of this study are available from the corresponding author upon reasonable request.

The included patients suffered a symptomatic CIAO in the intracranial ICA, middle cerebral artery (MCA), intracranial vertebral artery (VA), or basilar artery (BA). The symptoms included transient ischemic attack (TIA) and ischemic stroke within the past 90 days, which was attributable to hypoperfusion in the territory of the culprit arteries. Ischemic stroke referred to a new focal neurologic deficit lasting ≥24 h or lasting <24 h with new infarction on imaging, and TIA was defined as acute onset of a focal neurologic deficit lasting <24 h without new infarction on imaging. Hemodynamic impairment in the territory of the culprit artery was determined on imaging within 2 weeks before the procedure using any one of the following methods: (1) a cerebral blood flow (CBF) decrease of ≥30% when compared with the perfusion on the contralateral side for anterior circulation lesions or the anterior circulation territory for posterior circulation lesions on CTP; (2) an American Society of Interventional and Therapeutic Neuroradiology/Society of Interventional Radiology (ASITN/SIR) Collateral Flow Grading System score of <3 on digital subtraction angiography (DSA) (14); (3) hemodynamic ischemic lesion (defined as ischemic infarcts in the watershed distribution of the culprit arteries) by magnetic resonance imaging (MRI). All of the clinical and imaging data were reviewed centrally by the executive committee to decide whether the patient was eligible for enrollment criteria.

### CT Scanning and Processing of CT Perfusion Data

The imaging protocol includes whole-brain CTP before and after the endovascular recanalization. CTP studies were performed in the transverse plane by using a 256-slice axial CT scanner (GE Revolution CT) before and after the recanalization for all subjects. A 50-ml bolus of contrast media (Omnipaque, 350 mg I/ml; GE Healthcare, Shanghai, CN) was administered into an antecubital vein by using a power injector (Ulrich Injection System; Ulrich GMBH & CO. KG, Germany) with an injection rate of 5 ml/s. CT scanning was initiated 5 s after the start of the injection with the following acquisition parameters: 80 kV tube voltage, 150 mA, 5 mm slice thickness, 256 ^*^ 0.625 mm collimation, 0.5 s rotation time, 2.0 s cycle time, 25-cm field of view (FOV), 512 ^*^ 512 image matrix size, 32 slices. A total of 512 slices were obtained with a scan time of about 40 s and 160 mm scan length. Brain standard reconstruction was performed with the CT system. During the conduction of CTP, the mean arterial pressure of patients was generally maintained between 80 and 120 mmHg to avoid the impact of high or low blood pressure on CBF.

CTP source data were transferred from the clinical imaging database to a designated workstation (GE Workstation AW 4.7), and the whole-brain CTP images were reconstructed from source data for analysis. The software relies on the central volume principle to calculate perfusion parameters from the time–concentration curve. The mean transit time (MTT) was calculated by a closed-form deconvolution operation from the time–concentration curve and the arterial input function (AIF). The first artery to reach peak enhancement on the time–attenuation curve was selected as the AIF. The vein with the largest area under the time–attenuation curve was selected as the volume of fluid (VOF). The cerebral blood volume (CBV) was calculated from the areas under the time-concentration curves. The CBF was calculated according to the following equation: CBF = CBV/MTT. The time to peak (TTP) referred to the length of time for brain tissue to reach enhancement of peak density. Large vessels were automatically excluded via the brain perfusion software.

### Regions of Interest Selection

**Figure 1:**
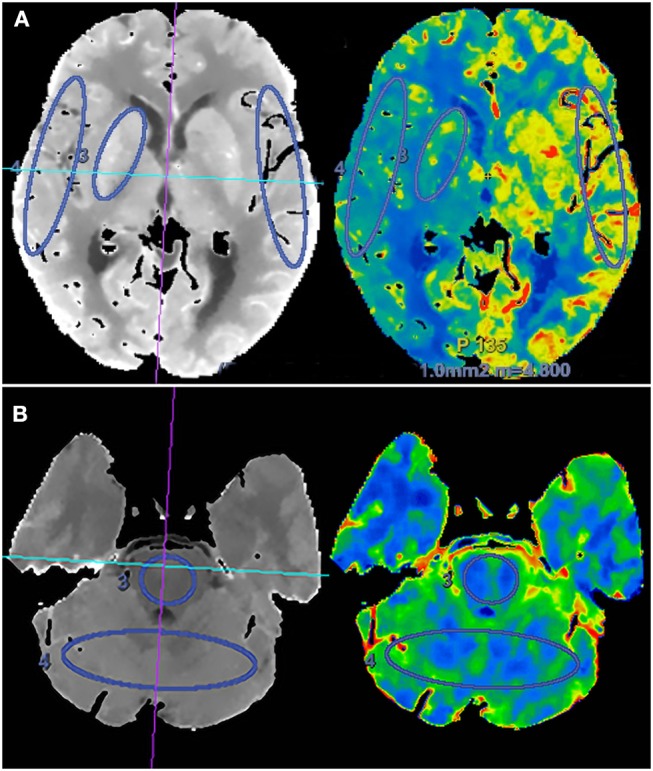
Schematic diagram of regions of interest (ROIs) in the region of perforating artery territory (PAT) and cortical artery territory (CAT) in anterior circulation **(A)** and posterior circulation **(B)**.

Two experienced neuroradiologists independently drew standardized elliptical regions of interest (ROIs) manually in the perforating artery territory (PAT) and cortical artery territory (CAT). For anterior circulation occlusion (ACO), PAT was selected in the basal ganglia, CAT was selected in the cortical middle cerebral artery territory, and mirror CAT was selected as reference area (Figure 1). For posterior circulation occlusion (PCO), PAT was selected in the pons, CAT was selected in the superior cerebellar artery territory, and the cortical middle cerebral artery territory was selected as reference area. The CTP absolute values in the regions of PAT, CAT, and reference areas in functional maps were measured (Figure 1). For each ROI, CBF, CBV, MTT, and TTP values were automatically calculated by the software. The relative CBF (rCBF), relative CBV (rCBV), relative MTT (rMTT), and relative TTP (rTTP) were obtained as follows: relative CTP values = absolute CTP values in PAT or CAT/absolute CTP values in reference ROIs. The proportion of rCBF change (rCBFc%) was defined as the mean value of rCBFc% in PAT and CAT, and it was obtained as follows: rCBFc% = (post-procedural rCBF–pre-procedural rCBF)/pre-procedural rCBF.

All the images of CTP were interpreted by two experienced neuroradiologists independently, and a CTP interpretation training program was performed for both of them to ensure the consistency of interpreting standard. The differences between their assessments were resolved by a third senior neuroradiologist (categorical data), or we took the average of their data (quantitative data) to reduce the impact of subjective factors.

### Periprocedural Management and Medical Treatment

The procedures were performed by experienced neurointerventionists. The operators were instructed to choose devices based on their experience and preference, as well as the conditions of individual patients. All patients were given a weight-based dose of 0.4–0.6 ml Fraxiparine (Sanofi Winthrop Industry) every 12 h subcutaneously for 3 days and monitored closely until discharge. All patients received aspirin (100 mg/day) and clopidogrel (75 mg/day) for more than 5 days before the operation. They were maintained on aspirin (100 mg/day) plus clopidogrel (75 mg/day) for 90 days after stenting. Other medical therapy included control of risk factors, such as blood pressure and low-density lipoprotein.

### Definition of Variable Subgroups

In stratified analysis, the patients were categorized into subgroups based on the occlusion sites, restenosis status, and time interval from revascularization to post-procedural CTP. Restenosis was defined as >70% stenosis within or immediately adjacent (within 5 mm) of the implanted stent compared with the diameter of reference vessels based on DSA or CT angiography source imaging. The short-term group was defined as those patients who underwent CTP within 7 days after the procedure, the mid-term group as CTP with a time interval from 7 days to 6 months, and the long-term group with a time interval of more than 6 months.

### Statistical Analysis

The statistical analysis was performed using a commercial statistical software package (SPSS for Windows, version 25.0, IBM-SPSS, Chicago, IL, US). The mean or median of CTP values on each ROI were calculated before and after the procedure. Normal distribution data were expressed as mean ± SD, while skew distribution data were expressed as median [interquartile range (IQR)], and categorical data were expressed as *n* (%). Differences in pre- and post-operative CTP values were assessed using paired *t*-test (normal distribution) or non-parametric test (skew distribution). The independent-samples *t*-test (normal distribution data) or non-parametric test was used for comparisons in rCBFc% between different groups (skew distribution data). Differences of *P* < 0.05 were considered statistically significant for two-tailed tests.

## Results

### Patient Baseline Characteristics

From June 2014 to June 2019, among 76 patients with symptomatic CIAO who have received endovascular treatment, 20 patients (56.1 ± 7.0 years old) with pre- and post-procedural CTP were recruited, including 16 male patients (80.0%) and four female patients (20.0%). The occlusion sites included intracranial ICA (*n* = 6), MCA (*n* = 7), intracranial VA (*n* = 5), and BA (*n* = 2). There were two patients with periprocedural complications (2/20, 10%), including one patient with perforator stroke that occurred immediately after the procedure and one patient with ischemic stroke due to in-stent subacute thrombosis that occurred 7 days after the procedure. There was no stroke, myocardial infarction, or death within 30 days after the procedure in the other 18 patients. The median length of the lesions was 13.0 mm. The median time interval from symptoms onset to the procedure and from documented total occlusion (confirmed by images) to the procedure was 60.5 and 19 days, respectively. The median time interval from pre-procedural CTP to the procedure and from the procedure to post-procedural CTP were 3 days and 4 months, respectively. Other baseline characteristics were shown in Table 1.

**Table 1:** Baseline clinical and endovascular treatment characteristics.

**Variable**	**Value**
Male, *n* (%)	16 (80%)
Age, mean ± SD	56.1 ± 7.0
BMI, median (IQR)	24.1 (23.3–27.2)
Risk factors	
Hypertension, *n* (%)	17 (85%)
Diabetes mellitus, *n* (%)	6 (30%)
Hyperlipidemia, *n* (%)	12 (60%)
Coronary heart disease, *n* (%)	2 (10%)
Smoking, *n* (%)	15 (75%)
Occlusion sites, *n* (%)	
Intracranial ICA	6 (30%)
MCA	7 (35%)
Intracranial VA	5 (25%)
BA	2 (10%)
Length of the lesion (mm), median (IQR)	13 (8.5–20.25)
Baseline NIHSS, median (IQR)	1 (0–2)
Baseline mRS, median (IQR)	1 (0–1)
Time interval from symptoms onset to the procedure (days), median (IQR)	60.5 (38.5–122.25)
Time interval from documented occlusion to the procedure (days), median (IQR)	19 (7.75–27.0)
Time interval from pre-operative CTP to revascularization (days), median (IQR)	3 (1–6)
Time interval from revascularization to post-operative CTP (months), median (IQR)	4.0 (0.2–23.7)

*BA, basilar artery; BMI, body mass index; CTP, CT perfusion; ICA, internal carotid artery; MCA, middle cerebral artery; VA, vertebral artery. Normal distribution data were expressed as mean ± SD, while skew distribution data were expressed as median (IQR, interquartile range). Categorical data were expressed as n (%)*.

### Post-procedural Hemodynamic Changes

The mean absolute CBF increased significantly after recanalization from 29.28 to 39.33 ml • 100 g^−1^ in PAT (*P* < 0.001) and from 32.60 to 47.84 ml • 100 g^−1^ in CAT (*P* < 0.001). The mean rCBF also increased significantly from 0.52 to 0.71 in PAT (*P* < 0.001) and from 0.59 to 0.85 in CAT (*P* < 0.001). The median and IQR of rCBFc% were 35.2 and 18.6–56.6%. There were significant decreases in MTT, TTP, rMTT, and rTTP after the procedure in both PAT and CAT, and the detailed values were shown in Table 2.

**Table 2:** Comparison of pre- and post-operative CTP values in the regions of PAT and CAT.

**CTP values**	**PAT**			**CAT**		
	**Pre-operation**	**Post-operation**	***P*-value**	**Pre-operation**	**Post-operation**	***P*-value**
CBF (ml·100 g^−1^·min^−1^)	29.28 ± 11.71	39.88 ± 10.66	<0.001	32.60 ± 11.06	47.84 ± 12.21	<0.001
CBV (ml·100 g^−1^)	2.57 (2.37–3.16)	2.55 (2.21–2.93)	0.073	3.50 ± 0.91	3.12 ± 0.61	0.024
MTT (s)	6.73 (5.03–9.08)	4.62 (3.31–5.78)	0.001	7.85 (6.09–9.93)	4.40 (3.61–5.70)	<0.001
TTP (s)	12.51 (11.75–14.21)	11.17 (10.33–12.81)	0.010	14.06 ± 2.23	11.93 ± 2.44	0.002
rCBF	0.52 ± 0.12	0.71 ± 0.19	<0.001	0.59 ± 0.10	0.85 ± 0.20	<0.001
rCBV	0.87 ± 0.14	0.83 ± 0.12	0.305	1.09 ± 0.24	1.01 ± 0.19	0.034
rMTT	1.66 (1.36–1.96)	1.08 (0.99–1.36)	0.002	1.85 (1.40–2.17)	1.13 (0.94–1.51)	<0.001
rTTP	1.20 (1.13–1.23)	1.06 (1.01–1.21)	0.001	1.31 ± 0.10	1.14 ± 0.13	<0.001

*PAT, perforating artery territory; CAT, cortical artery territory; CBF, cerebral blood flow; CBV, cerebral blood volume; MTT, mean transit time; TTP, time to peak; rCBF, relative cerebral blood flow; rCBV, relative cerebral blood volume; rMTT, relative mean transit time; rTTP, relative time to peak. Normal distribution data were expressed as mean ± SD, while skew distribution data were expressed as median (IQR, interquartile range)*.

### Subgroup Analysis

**Figure 2:**
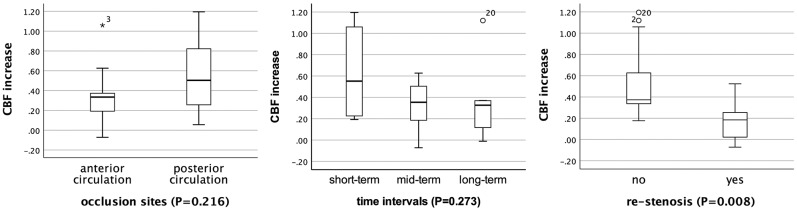
Comparison of the proportion of relative cerebral blood flow change (rCBFc%) in patients with different occlusion sites, at different time intervals, and restenosis status. *Represents extreme value (more than three box lengths from either end of the box), and circles represent outliers (between one and a half and three box lengths from either end of the box).

In subgroup analysis, the rCBF increase retained statistically significant in patients with different occlusion sites, at different time intervals, and in patients without restenosis (*P* < 0.05; Table 3). For patients with short-term follow-up (55.2%), the rCBFc% was relatively higher than that in patients with mid-term (35.4%) and long-term follow-up (32.7%), although without statistical difference (*P* = 0.273; Figure 2). For patients with restenosis, the rCBF also showed a tendency to increase without a statistical difference (Table 3), but the rCBFc% was significantly lower than that in patients without restenosis (18.5 vs. 37.3%, *P* = 0.008; Figure 2). The rCBFc% in patients with posterior circulation occlusion was relatively higher than that in patients with anterior circulation occlusion, although there was no statistical difference (50.4 vs. 33.4%, *P* = 0.216; Figure 2).

**Table 3:** Stratified analysis of pre- and postoperative comparison of rCBF in the regions of PAT and CAT.

		**PAT**			**CAT**		
		**Pre-operation**	**Post-operation**	***P*-value**	**Pre-operation**	**Post-operation**	***P*-value**
Occlusion sites	ACO (*n* = 13)	0.54 ± 0.13	0.70 ± 0.19	0.001	0.59 ± 0.10	0.84 ± 0.23	0.001
	PCO (*n* = 7)	0.48 ± 0.11	0.73 ± 0.20	0.028	0.59 ± 0.09	0.88 ± 0.15	0.006
Time interval	Short-term (*n* = 6)	0.55 ± 0.06	0.85 ± 0.18	0.024	0.63 ± 0.10	1.01 ± 0.13	0.006
	Midterm (*n* = 6)	0.59 ± 0.14	0.72 ± 0.18	0.049	0.57 ± 0.12	0.84 ± 0.27	0.011
	Long-term (*n* = 8)	0.44 ± 0.11	0.60 ± 0.14	0.016	0.58 ± 0.08	0.74 ± 0.10	0.014
Restenosis	Yes (*n* = 7)	0.50 ± 0.13	0.56 ± 0.16	0.161	0.57 ± 0.10	0.71 ± 0.23	0.084
	No (*n* = 13)	0.53 ± 0.12	0.79 ± 0.15	<0.001	0.60 ± 0.10	0.93 ± 0.14	<0.001

*PAT, perforating artery territory; CAT, cortical artery territory; ACO, anterior circulation occlusion; PCO, posterior circulation occlusion; time interval, short-term referred to CT perfusion (CTP) reexamination within 7 days after the procedure, midterm referred to CTP reexamination between 3 and 6 months after the procedure, and long-term referred to CTP reexamination at least 6 months after the procedure; rCBF, relative cerebral blood flow. Restenosis was defined as >70% stenosis compared with the diameter of reference vessels. Normal distribution data were expressed as mean ± SD, while skew distribution data were expressed as median (IQR, interquartile range)*.

## Discussion

To the best of our knowledge, this is the first conducted study focusing on post-procedural hemodynamic changes over time in patients with CIAO based on CTP. CTP is a readily accessible and reliable method for the evaluation of cerebral hemodynamic characteristics and has been widely used in the cerebral hemodynamic evaluation of stenotic or occlusive cerebrovascular disease in clinical practice (15–20). In this study, CTP parameters (especially CBF) were used to evaluate the hemodynamic changes over time after successful recanalization of CIAO.

The results indicated that in patients with CIAO who had received endovascular recanalization, post-procedural CBF increased significantly in both PAT and CAT regions. The stratified analysis demonstrated that the CBF increase retained statistically significant in patients with different occlusion sites, at different time intervals, and in patients without restenosis. This indicated that successful recanalization might increase CBF and improve cerebral hemodynamics in patients with CIAO. Similar changes had been found in patients with chronic carotid artery occlusion after interventional or surgical recanalization in previous studies (11).

In the present study, the CBF increase retained relatively stable in the long term after successful recanalization, although the mid-term tendency of decrease was observed. The short-term CBF increase may be attributed to the hyperperfusion phenomenon after successful recanalization. The CBF values in the midterm and long term were lower than that in the short term, and this reflected the real and meaningful CBF changes after the recanalization, and it can keep relatively stable over time, which suggested that the long-term efficacy of endovascular treatment for CIAO was acceptable.

For patients with restenosis, the post-procedural CBF also showed a tendency to increase without statistical difference compared with pre-procedural CBF, but the rCBFc% was significantly lower than that in patients without restenosis (18.5 vs. 37.3%, *P* = 0.008). This indicated that in patients with restenosis, the CBF tends to decrease, even though the patient remains asymptomatic.

The rCBFc% in patients with posterior circulation occlusion was relatively higher than that in patients with anterior circulation occlusion (50.4 vs. 33.4%, *P* = 0.216). However, due to the insignificant statistical difference and the difference in baseline rCBF between the anterior and posterior circulation occlusion in the regions of PAT (0.48 vs. 0.54), we cannot conclude currently whether patients with CIAO at posterior circulation benefit more from successful recanalization than those with CIAO at anterior circulation, which needs to be confirmed in future studies with a larger sample size.

In the present study, most of the patients had a good outcome during the follow-up period, and recurrent post-operative stroke occurred only in two patients. Therefore, we did not evaluate whether improved hemodynamics was associated with good outcomes or reduced risk of recurrent stroke. Future studies with larger sample sizes and longer follow-up intervals are needed to confirm this relationship.

Potential limitations of this study should be mentioned. First, the sample size was small in this study, and the patients were enrolled from a single center, so potential selection bias may be inevitable. Second, ROIs in this study were drawn manually, and only parts of the PAT or CAT were evaluated. It is practically challenging to draw ROIs in a completely consistent area before and after the recanalization. However, we had tried our best to draw almost the same ROIs in the same areas before and after the recanalization. Third, the retrospective nature of the study necessitates further studies to confirm our conclusions.

## Conclusion

In patients with symptomatic CIAO, the CBF may increase and be relatively stable over time after successful recanalization except for restenosis.

## Data Availability Statement

The data that support the findings of this study are available from the corresponding author upon reasonable request.

## Ethics Statement

The study was performed according to the guidelines from the Helsinki Declaration, and it was approved by the IRB of the hospital. Written informed consent was obtained from all patients or their legally authorized guardian.

## Author Contributions

KK and BY contributed to the conception/design of the study, the acquisition, analysis, and interpretation of data, drafting of the manuscript, and final approval of the version to be published. XG, WG, and GM were responsible for the acquisition of data and final approval of the version to be published. XC was responsible for critical revision of the manuscript and final approval of the version to be published. NM, XZ, and ZM were responsible for the conception/design of the study, revision of the work, final approval of the version to be published, and agreement to be accountable for all aspects of the work.

## Conflict of Interest

The authors declare that the research was conducted in the absence of any commercial or financial relationships that could be construed as a potential conflict of interest. The handling editor declared a past co-authorship with one of the authors XZ.
